# Antioxidant Melatonin: Potential Functions in Improving Cerebral Autoregulation After Subarachnoid Hemorrhage

**DOI:** 10.3389/fphys.2018.01146

**Published:** 2018-08-17

**Authors:** Zhen-Ni Guo, Hang Jin, Huijie Sun, Yingkai Zhao, Jia Liu, Hongyin Ma, Xin Sun, Yi Yang

**Affiliations:** ^1^Department of Neurology, The First Hospital of Jilin University, Changchun, China; ^2^Clinical Trial and Research Center for Stroke, Department of Neurology, The First Hospital of Jilin University, Changchun, China; ^3^Cadre Ward, The First Hospital of Jilin University, Changchun, China; ^4^Shenzhen Institutes of Advanced Technology, Chinese Academy of Sciences, Shenzhen, China

**Keywords:** melatonin, cerebral autoregulation, subarachnoid hemorrhage, antioxidant, sympathetic nerve, endothelial function

## Abstract

Subarachnoid hemorrhage (SAH) is a subtype of stroke with high mortality and morbidity. Impaired cerebral autoregulation following SAH has been reported owing to effects on sympathetic control, endothelial function, myogenic response, and cerebral metabolism. Impaired cerebral autoregulation is associated with early brain injury, cerebral vasospasm/delayed cerebral ischemia, and SAH prognosis. However, few drugs have been reported to improve cerebral autoregulation after SAH. Melatonin is a powerful antioxidant that is effective (easily crosses the blood brain barrier) and safe (tolerated in large doses without toxicity). Theoretically, melatonin may impact the control mechanisms of cerebral autoregulation via antioxidative effects, protection of endothelial cell integrity, suppression of sympathetic nerve activity, increase in nitric oxide bioavailability, mediation of the myogenic response, and amelioration of hypoxemia. Furthermore, melatonin may have a comprehensive effect on cerebral autoregulation. This review discusses the potential effects of melatonin on cerebral autoregulation following SAH, in terms of the association between pharmacological activities and the mechanisms of cerebral autoregulation.

## Introduction

Cerebral autoregulation is defined as the mechanism by which constant cerebral blood flow is maintained, despite changes in arterial blood pressure (Guo et al., 2016). In the cerebral arterial system, cerebral autoregulation has been reported to be involved in all types of stroke and is related to secondary brain injury and prognosis (Vavilala et al., 2003; Chen et al., 2014b; Guo et al., 2014; Ma et al., 2016). In the reviews of Paulson and Strandgaard in 1984 and 1990, they concluded that the regulating mechanisms of cerebral autoregulation are including sympathetic control, cerebral metabolism, endothelial function and myogenic response (Strandgaard and Paulson, 1984; Paulson et al., 1990). Later, Bailey proposed that oxidative stress is also associated with impaired cerebral autoregulation and blood-brain barrier leakage (Bailey et al., 2011).

Subarachnoid hemorrhage (SAH) is a subtype of stroke with high mortality and significant morbidity. Delayed cerebral vasospasm and delayed cerebral ischemia are among the primary causes of poor prognosis following SAH. Cerebral autoregulation has been reported to be impaired after SAH, and this phenomenon is associated with cerebral vasospasm/delayed cerebral ischemia (Budohoski et al., 2012, 2013; Otite et al., 2014; Calviere et al., 2015; Guo et al., 2016; Santos et al., 2016; Gaasch et al., 2018). Thus, cerebral autoregulation may be a potential therapeutic target for improving prognosis after SAH.

Melatonin is a hormone secreted by the pineal gland during the dark phase of the light-dark cycle, which is modulated by light-dark cycle (Bruls et al., 2000). Besides the pineal gland, melatonin was also produced in bone marrow (Tan D.X. et al., 1999). In addition, Tan D. et al. (1999) found high levels of melatonin in the bile of mammals of unknown origin. Previous studies reported that melatonin is a powerful antioxidant, which is known to be effective (it easily crosses the blood brain barrier) and safe (non-toxic in high doses) (Reiter et al., 2000). It has been studied in several cerebrovascular diseases, including ischemic stroke (Beker et al., 2015; Feng et al., 2017), intracerebral hemorrhage (Li et al., 2009; Lekic et al., 2010), and SAH (**Table 1**) (Fang et al., 2009; Wang et al., 2012, 2013; Chen et al., 2014a,c, 2015; Dong et al., 2016; Zhao et al., 2016), with respect to the mechanisms of antioxidation (Garcia et al., 2014; Zhang and Zhang, 2014; Manchester et al., 2015) and anti-inflammation (Agil et al., 2013; Mauriz et al., 2013; Chen et al., 2014a; Yang et al., 2014; Liu et al., 2015; Dong et al., 2016). These pharmacological activities of melatonin also potentially improve cerebral autoregulation after SAH. The present review discusses the potential effect of melatonin on cerebral autoregulation after SAH with respect to the association between pharmacological activities and mechanisms regulating cerebral autoregulation.

**Table 1:** Functions of melatonin in improving brain injury after subarachnoid hemorrhage.

Journal	First author	Year	Action targets
J Pineal Res	Dong Y	2016	Regulating NLRP3 inflammasome and apoptosis signaling.
Mol Neurobiol	Zhao L	2016	Regulating melatonin receptor/Sirt1/NF-κB signaling pathway
J Pineal Res	Chen J	2014	Regulation of pro-inflammatory cytokines
J Pineal Res	Chen J	2014	Regulating mitochondrial pathway
J Pineal Res	Wang Z	2013	Regulating TLR4-mediated inflammatory pathway
J Pineal Re	Wang Z	2012	Activating the Nrf2-ARE pathway
Mediators Inflamm	Fang Q	2009	Regulating nuclear factor-kappa pathway and proinflammatory cytokines expression


## Cerebral Autoregulation Dysfunction After SAH

### Clinical Findings

Recently, an increasing number of studies have focused on the relationship between cerebral autoregulation and SAH and have reported that the impairment of cerebral autoregulation is related to poor prognosis after SAH. Otite et al. (2014) reported that patients who developed delayed cerebral vasospasm and delayed cerebral ischemia after SAH had worse cerebral autoregulation than did those who did not develop either of the conditions. Budohoski et al. (2015) conducted a study to determine the underlying consequences of unilateral and bilateral cerebral autoregulation damage on outcomes in SAH patients. They found that unilateral and bilateral cerebral autoregulation damage was related to delayed cerebral ischemia and unfavorable outcomes, respectively (Budohoski et al., 2015). Santos et al. (2016) analyzed the pathophysiological basis of the impairment of cerebral autoregulation in SAH and its relationship to prognosis. They found that cerebral autoregulation was significantly impaired in SAH patients who developed delayed cerebral ischemia compared with those who did not develop secondary brain injury or cerebral vasospasm alone (Santos et al., 2016). Similar results were reported by several studies (**Table 2**) (Lang et al., 2001; Soehle et al., 2004; Tseng et al., 2006; Budohoski et al., 2012, 2013, 2016; Calviere et al., 2015; Gaasch et al., 2018).

**Table 2:** Literature on cerebral autoregulation (CA) and subarachnoid hemorrhage in humans.

Journal	First author	Year	Main outcomes
Crit Care Med	Gaasch M	2018	CA was associated with delayed cerebral ischemia (DCI) and poor functional outcome
Neurology	Santos GA	2016	CA can predict neurologic complications
Acta Neurochir Suppl	Budohoski KP	2016	Impaired CA in the first 5 days after SAH is predictive of DCI
Neurocrit Care	Calviere L	2015	Early deterioration of CA was strongly predictive of DCI
Neurocrit Care	Budohoski KP	2015	Unilateral CA failure was seen in patients who developed DCI, and bilateral CA failure was seen more frequently in patients with unfavorable outcome
Stroke	Otite F	2014	Impaired CA is associated with vasospasm and DCI
J Cereb Blood Flow Metab	Budohoski KP	2013	CA can aid in predicting DCI
Stroke	Budohoski KP	2012	Disturbed CA in the first 5 days after SAH significantly increases the risk of DCI
Neurosurg Focus	Tseng MY	2006	CA may help identify patients at high risk of delayed ischemic neurological deficits.
Anesth Analg	Soehle M	2004	CA was impaired during cerebral vasospasm
Crit Care Med	Lang EW	2001	CA impairment precedes vasospasm, and ongoing vasospasm worsens CA


### Mechanisms

Impaired cerebral autoregulation after SAH is possibly caused by oxidative stress, endothelial dysfunction, sympathetic activation, myogenic response disorder, and abnormal cerebral metabolism. A detailed study of these mechanisms might lead to future therapeutic possibilities.

#### Oxidative Stress After SAH

**FIGURE 1:**
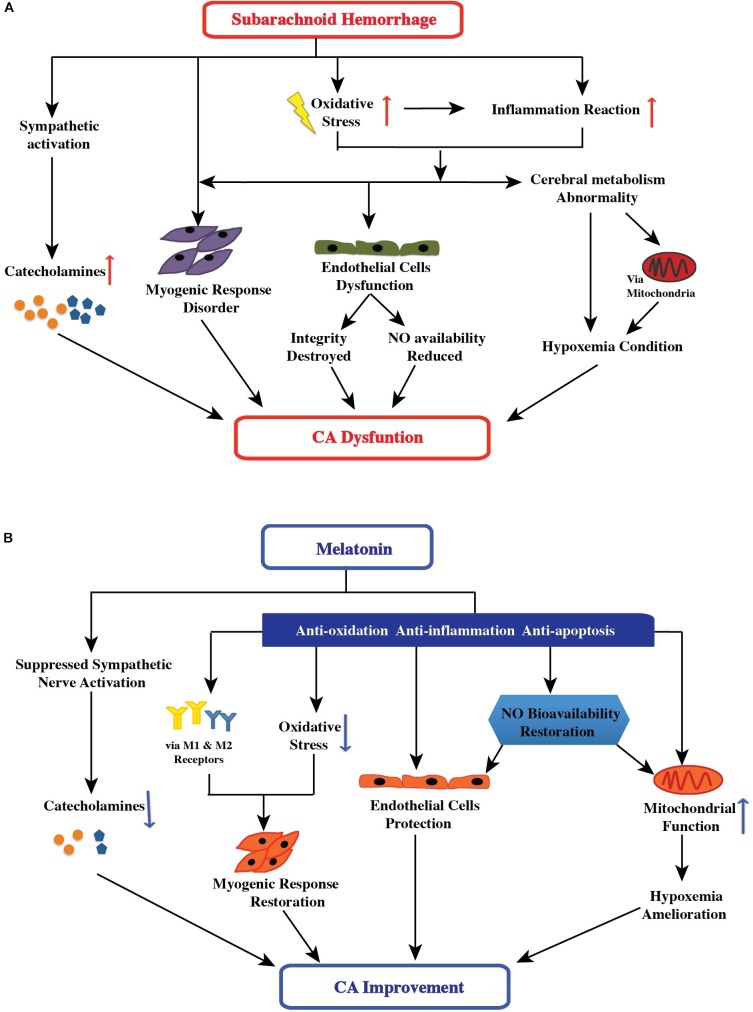
**(A)** Effect of subarachnoid hemorrhage on the control mechanisms of cerebral autoregulation. After subarachnoid hemorrhage, oxidative stress is implicated in the etiology of all stages: the high free-radical concentration leading to impaired cerebral autoregulation has direct and indirect effects, including changing smooth muscle tone, damaging endothelial cell function (followed by destruction of endothelial cell integrity and reduction of nitric oxide availability), and inducing an inflammatory response (followed by endothelial cell dysfunction and hypoxemia). Additionally, sympathetic activation and abnormal cerebral metabolism after SAH aggravated the impairment of cerebral autoregulation. **(B)** Possible therapeutic targets of melatonin in improving cerebral autoregulation after subarachnoid hemorrhage. After subarachnoid hemorrhage, melatonin may potentially impact the control mechanisms of cerebral autoregulation via suppression of sympathetic nerve and antioxidant activity, increase in nitric oxide bioavailability, direct and indirect mediation of the myogenic response, and amelioration of hypoxemia. Furthermore, melatonin can have a comprehensive effect on cerebral autoregulation.

After SAH, oxidative stress is implicated in the etiology of at all stages of SAH (early brain injury, cerebral vasospasm, and delayed cerebral ischemia) (Ersahin et al., 2010; Zhang et al., 2015; Li et al., 2016; Ye et al., 2018). The high concentration of reactive oxygen species (ROS)/reactive nitrogen species (RNS) is considered to be associated with impaired cerebral autoregulation (Choi et al., 2001; Shin et al., 2002) (**Figure 1**). One important mechanism has been reported to result in impaired cerebral autoregulation is because of the direct and indirect actions of ROS/RNS on K+ channels. The K+ channels, including ATP-sensitive K+ channels and large conductance Ca^2+^-activated K^+^ channels, can regulate the activation and contraction of cerebral arterial muscle cells, and subsequently change the smooth muscle tone (Lee et al., 1993; Nelson and Quayle, 1995; Shin and Hong, 2004; Zagorac et al., 2005). Moreover, the high concentration of free radicals may cause impaired cerebral autoregulation in several other pathways, including damaged endothelial cells function (followed by integrity destroyed and nitric oxide availability reduced), and inducted inflammatory response (followed by endothelial cells dysfunction and hypoxemia condition). These factors are discussed in the following sections (**Figure 1**).

#### Endothelial Dysfunction After SAH

The vascular endothelial mechanism is an essential part of cerebral autoregulation because endothelial cells modulate many aspects of vascular functioning, particularly in controlling the vascular tone (Pries et al., 2000; Rodella et al., 2013). The structural and functional integrity of endothelial cells is essential for maintaining stable cerebral autoregulation (Preckel et al., 1996; White et al., 2000; Ainslie et al., 2007; Guo et al., 2016). After SAH, both structural and functional integrity were damaged because of factors, such as the high concentration of ROS/RNS and inflammatory responses (**Figure 1**) (Kajita et al., 1998; Scharbrodt et al., 2009; Szatmari et al., 2010; Sabri et al., 2011; Qin et al., 2012; Liu et al., 2016; de Azevedo et al., 2017; Shekhar et al., 2017; Armstead et al., 2018). In inflammatory responses, various inflammatory pathways, such as the NF-κB pathway (Pawlowska et al., 2018), NLRP3 pathway (Li et al., 2016; Shao et al., 2016), and TLR4 pathway (Zhang et al., 2016), are activated and have negative effects on the arterial endothelium after SAH. In the downstream of these pathways are inflammatory factors, interleukin-1β, and tumor necrosis factor-α. These inflammatory factors act on vascular endothelium, resulting in changes in the concentration and bioavailability of endothelium-derived nitric oxide.

Nitric oxide, the most important vasodilation factor, can regulate the vascular tone of small arteries; the mechanism is that nitric oxide diffuses into the adjacent smooth muscle cells and relaxes them by increasing cyclic guanosine monophosphate (Kajita et al., 1998). Because of the physiological effects of nitric oxide, Guo et al. (2016) proposed that reduced nitric oxide availability may relate to the impaired cerebral autoregulation after SAH due to endothelium-dependent mechanism. The results reported by Tseng et al. (2005) support this hypothesis; these authors found that pravastatin, a member of the drug class of statins, can improve vascular endothelium-dependent relaxation to acetylcholine and increase endothelial nitric oxide synthase activity, as well as improve cerebral autoregulation after SAH (Tseng et al., 2005; Yamamoto et al., 2007). Thus, these studies indicated that the improvement in endothelial function is a therapeutic target to improve cerebral autoregulation.

#### Sympathetic Activation After SAH

The cerebrovascular bed is innervated by sympathetic nerve fibers (Edvinsson et al., 1975; Hamner et al., 2010). The sympathetic nervous system regulates cerebral blood flow by managing cerebral vascular resistance (Aubineau et al., 1980; Busija, 1985; Guo et al., 2016). Theoretically, after the stimulation of sympathetic nervous system, alpha-1 adrenergic receptors are activated by norepinephrine released by post-ganglionic sympathetic neurons, resulting in vasoconstriction (Aubineau et al., 1980; Busija, 1985; Guo et al., 2016). Several studies have reported that sympathetic control plays an important role in regulating cerebral autoregulation, and that sympathetic dysfunction can cause impaired cerebral autoregulation (Sadoshima et al., 1985; Hamner and Tan, 2014; Guo et al., 2016). Notably, the acute stage of SAH is accompanied by significant sympathetic activation (Moussouttas et al., 2012b, 2014). Sympathetic activation results in increased concentration of circulating catecholamines (epinephrine, noradrenaline, and serotonin), which are associated with cerebral vasospasm and delayed cerebral ischemia after SAH (Grad et al., 1991; Dilraj et al., 1992; Naredi et al., 2000; Banki et al., 2005; Moussouttas et al., 2012a). The vasoconstriction of blood vessels caused by sympathetic activation after SAH is a possible mechanism underlying cerebral autoregulation dysfunction (**Figure 1**).

#### Myogenic Response Disorder After SAH

Smooth muscle is a main component of cerebral arteries. The control of arterial myogenic tone was first described by Bayliss (1902). The myogenic response was regulated by a complex mechanism, and some of these mechanisms are out of balance after SAH (Lidington et al., 2018). (1) Previous study reported that potassium channels are important regulators of vascular tone. SAH can reduce potassium currents in cerebral artery smooth muscle cells and then enhanced constriction (Jahromi et al., 2008). (2) After SAH, endothelial dysfunction was observed, resulting in reduced vasodilating factors levels. Thus, it is reasonable to speculate that endothelial dysfunction augments myogenic response disorder (Lidington et al., 2018). (3) The effect of ROS on myogenic response disorder was our concern. ROS are believed to be involved in cellular signaling in blood vessels, and to directly and indirectly mediate vascular smooth muscle via regulating endothelium-dependent contractions pathway (Cosentino et al., 1994), and calcium-activated potassium channels (Wei et al., 1996; Faraci, 2006). Previous studies also reported that both relaxation and contraction of the vascular muscle were caused by ROS, which may dependent on concentrations (Faraci, 2006). For instance, Rosenblum found that the generation of superoxide using acetaldehyde and xanthine oxidase produces dilation of cerebral arterioles at low substrate concentrations, but vasoconstriction at higher substrate concentrations followed by dilation (Rosenblum, 1983). Studies *in vivo* found that hydrogen peroxide acts as a vasodilator on small cerebral arteries via activated potassium channels. However, high concentrations of hydrogen peroxide can produce vasoconstriction followed by vasodilation (Faraci, 2006). The general trend is that, ROS produces vasodilation at low concentrations and vasoconstriction at higher concentrations (Rosenblum, 1983; Cosentino et al., 1994; Wei et al., 1996; Faraci, 2006). Thus, high concentration of ROS (derived from blood) may augment the myogenic tone via directly and indirectly mediate vascular smooth muscle after SAH. Recently, a study from Deng et al. (2018) provided direct evidence. They studied the effects of extravascular hemolyzed blood on arteriolar myogenic constriction and found that extravascular hemolyzed blood augments the myogenic constriction of cerebral arterioles, possibly by increasing the vascular production of superoxide. In addition to ROS pathways, a study reported that tumor necrosis factor-α/sphingosine-1-phosphate signaling can augment the myogenic tone in experimental SAH mouse model (Yagi et al., 2015). These studies indicated that the effects of the myogenic response on cerebral autoregulation may be caused multiple pathways and need further analysis.

#### Metabolism Abnormal Impaired Cerebral Autoregulation After SAH

Under physiological conditions, when the cerebral blood volume was decreased, some vasoactive substances were released from the brain, caused the cerebral arteries to become dilated, and vice versa. This phenomenon was considered a metabolic mechanism of cerebral autoregulation to maintain stable cerebral perfusion. Actually, apart from vasoactive substances, oxygen and carbon dioxide levels and metabolites can regulate cerebral autoregulation. After SAH, the reduced brain tissue oxygen pressure and brain pH (Carvi y Nievas et al., 2005) can change cerebral microcirculation and metabolism, perhaps partly because of oxidative damage (Lopez et al., 2009). These changes may cause cerebral autoregulation dysfunction (**Figure 1**).

## The Role of Melatonin in Improving Cerebral Autoregulation After SAH

### Melatonin Improves Cerebral Autoregulation by Antioxidation

Previous studies have found that melatonin and its metabolites (mainly *N*^1^-acetyl-*N*^2^-formyl-5-methoxykynuramine and *N*-acetyl-5-methoxykynuramine) are powerful free radical scavengers, and scavenge various types of free radicals, such as hydroxyl radicals and hydrogen peroxide. Through a cascade reaction involving melatonin and its metabolites, a melatonin molecule can scavenge up to 10 ROS/RNS (Tan et al., 2002; Zavodnik et al., 2006; Hardeland et al., 2007; Tan et al., 2007). Besides, Boussard et al. (2006) reported that the third melatonin binding site (MT3), characterized as the enzyme quinone reductase 2, may contribute to melatonin antioxidant properties by inhibiting the electron transfer reactions of quinones (Boussard et al., 2006; Pandi-Perumal et al., 2008; Emet et al., 2016).

In addition, melatonin plays an important role in activating antioxidant defenses. Venkataraman et al. (2010) found that exogenous melatonin supplementation can rescue the decreased mRNA expression of Cu/Zn superoxide dismutase and glutathione peroxidase-4 in a polychlorinated biphenyl-induced neuronal damage rat model. Akcay et al. (2005) reported that treatment with exogenous melatonin can maintain malondialdehyde levels and catalase and superoxide dismutase activities at normal levels in the brain cortex of a kainic acid-induced injury rat model. Moreover, melatonin can inhibit pro-oxidant enzymes, such as inducible nitric oxide synthase (Lopez et al., 2006; Kang et al., 2013).

Thus, melatonin is a useful antioxidant, and acts via multiple antioxidant pathways; its antioxidative actions directly and indirectly improve cerebral autoregulation by protecting endothelial function and increasing nitric oxide bioavailability, mediating myogenic responses, and ameliorating conditions of hypoxemia. This is discussed in the following sections.

### Melatonin Improves Cerebral Autoregulation by Protecting Endothelial Function and Increasing Nitric Oxide Bioavailability

As mentioned above, melatonin serves as an antioxidant via several pathways. It also has anti-inflammatory effects. Several studies have reported the possible pathways through which melatonin attenuates inflammation in the brain (Carrascal et al., 2018; Wang et al., 2018). Jumnongprakhon et al. (2016) found that melatonin can prevent methamphetamine-induced inflammatory responses by inhibiting the nuclear factor-κB pathway and promoting the nuclear factor erythroid 2-related factor-2 pathway before blood-brain barrier impairment. Dong et al. (2016) suggested that melatonin regulates the NLRP3 inflammasome pathway, and thus attenuates early brain injury after SAH. Wang et al. (2013) proposed that melatonin can alleviate secondary brain damage through the TLR4-mediated inflammatory pathway after SAH. Fu et al. (2017) also reported that melatonin supplementation may be a valuable therapeutic strategy in cases of inflammatory neurological dysfunction, and that melatonin may subserve this function through the inhibition of TLR4 signaling. Zhao et al. (2015) reported that melatonin attenuates sepsis-induced brain injury by activating silent information regulator 1 signaling (Zhao et al., 2015). In addition, oxidative stress plays a vital role in mediating the initial phase of the inflammatory reaction by regulating leukocyte recruitment and maturation and activating intracellular inflammatory pathways, resulting in increased levels of various inflammatory mediators (Cristofanon et al., 2009; Radogna et al., 2010). Melatonin can regulate signaling through these pathways and thus inhibit inflammatory processes (**Figure 1**).

Previous studies have reported that melatonin functions to increase nitric oxide bioavailability. Aladag et al. (2009) conducted a study in an SAH rat model and showed that the administration of melatonin ameliorates cerebral vasospasm via an increase in serum nitric oxide concentration and a decrease in the levels of arginase and oxidative stress in the brain. Similarly, using intermittent hypoxia rat models, Tjong et al. (2008) showed that melatonin ameliorates constitutive nitric oxide production and large conductance calcium-activated potassium channel activity through an antioxidant pathway. Wakatsuki et al. (2001) studied the antioxygenation effect of melatonin on the oxidized low-density lipoprotein-induced impairment of nitric oxide production, and found that pre-treatment with melatonin reversed the oxidized low-density lipoprotein-induced reduction in nitric oxide production. In their review, Simko and Paulis noted that melatonin may increase nitric oxide levels via the promotion of nitric oxide production and/or the prevention of coupling to the superoxide anion radical (Simko and Paulis, 2007). However, some studies have reported that melatonin reduces nitric oxide levels in middle cerebral artery occlusion stroke rat models (Pei et al., 2003) and cerebral ischemia/reperfusion Mongolian gerbil models (Guerrero et al., 1997). Thus, the role of melatonin in nitric oxide production requires further investigation.

Previous studies show that melatonin can act as an endothelial protective agent via the disruption of oxidative stress and inflammatory response pathways and may also regulate nitric oxide concentration and bioavailability. Thus, it can protect the integrity and function of vascular endothelial cells.

### Melatonin Improves Cerebral Autoregulation by Suppressing Sympathetic Nerve Activity

In previous studies, melatonin has been shown to regulate sympathetic nerve activity. Viswanathan et al. (1986) found that the administration of melatonin to Syrian hamsters suppressed the sympathetic nervous system. Cagnacci et al. (1998) found that the administration of melatonin decreased blood pressure and blunt noradrenergic activation in young women. Arangino et al. (1999) reported that oral administration of melatonin could reduce blood pressure, vascular reactivity, and norepinephrine levels in men. Girouard et al. (2004) found that exogenous melatonin improved the baroreflex response associated with improved antioxidation in spontaneously hypertensive rats, suggesting a correlation between antioxidation and the decreased sympathetic tone induced by melatonin. In another study of spontaneously hypertensive rats, K-Laflamme et al. (1998) found that after 20 min of melatonin administration, the plasma epinephrine concentration reduced by approximately 60%, and the norepinephrine concentration decreased by approximately 30%. This indicated that the action of melatonin involved the inhibition of basal sympathoadrenal tone (K-Laflamme et al., 1998). Interestingly, Olmez and Kurcer (2003) found that melatonin can attenuate alpha-adrenergic-induced contractions by increasing vasoactive intestinal peptide levels in isolated rat penile bulbs. In addition, several studies have found that melatonin can affect the neural control of reflex changes in muscles and sympathetic nerve activity in the skin (Ray, 2003; Muller et al., 2013). Hence, the role of melatonin in regulating sympathetic nerve activity is gradually becoming clearer. However, these evidences of melatonin in regulating sympathetic nerve activity is based on systemic effects, we also tried to find direct evidence of melatonin on cerebral regulation. After careful searching, only one study was found. Bang et al. (2012) reported that exogenous melatonin did not affect the cardiovascular reflex and dynamic cerebral autoregulation responses to acute hypotension in twelve healthy men. The reason for the negative results may due to the subjects were healthy adults and the sample size was too small. Thus, theoretically, melatonin may be a potentially useful drug for improving cerebral autoregulation via a reduction in sympathetic nerve activity after SAH (**Figure 1**), but the actual effect of melatonin remains to be studied.

### Melatonin Improves Cerebral Autoregulation by Mediating Myogenic Response

As mentioned in the previous section, the ROS concentration may play an important role in regulating vasomotor function after SAH. Melatonin acts as a powerful ROS scavenger, functioning to mediate myogenic responses after SAH. In addition, exogenous melatonin reduces the concentration of tumor necrosis factor-α in the brain (Pazar et al., 2016; Taniguti et al., 2018), and may thus reduce tumor necrosis factor-α-mediated myogenic tone augmentation (Yagi et al., 2015). Weekley (1991) reported that melatonin induces the dose-dependent relaxation of precontracted vascular smooth muscle of rat aorta, and this response was not affected by vascular endothelium removal.

Furthermore, melatonin can have direct effects on smooth muscle through its receptors. Humans have two plasma membrane receptors of melatonin, MT1 and MT2, which are expressed in various tissues, including brain, retina, cardiovascular system, and liver tissues (Ekmekcioglu, 2006). MT1 and MT2 belonging to the G-protein-coupled receptor superfamily, which constitutes adenylate cyclase inhibition by binding to various G-proteins (Pandi-Perumal et al., 2008; Emet et al., 2016). In the central nervous system in humans, melatonin receptors are observed in suprachiasmatic nuclei (Weaver and Reppert, 1996), retina (Reppert et al., 1995; Thomas et al., 2002; Ekmekcioglu, 2006), hippocampus (Savaskan et al., 2002; Savaskan et al., 2005), and cerebellar cortex (Al-Ghoul et al., 1998). In studies on the caudal artery, the MT1 receptor mRNA was primarily found in the smooth muscle layer, whereas the MT2 receptor mRNA appeared more evenly distributed throughout the vessel wall. However, both MT1 and MT2 in vascular smooth muscle cells can regulate the vascular tone. Doolen et al. (1998) indicated that MT1 receptor activation may mediate vasoconstriction. Subsequently, Lew and Flanders (1999) further indicated that melatonin elicited the contraction of the rat tail artery by activating an MT1 receptor that coupled to the activated L-type calcium channels. For the MT2 receptor, study conclusions are inconsistent. Masana et al. (2002) indicated that after using MT2 antagonists, the melatonin-mediated vasocontraction was enhanced, indicating MT2 receptors located in vascular smooth muscle mediate vasodilation. Similarly, Doolen et al. (1998) also found MT2 receptors may induce relaxation. However, a study reported that MT2 receptor activation in coronary vascular smooth muscle cells is associated with inhibiting nitric oxide-induced increases in cyclic GMP and coronary arterial relaxation (Tunstall et al., 2011). The comprehensive effects of melatonin receptors in regulating the myogenic response warrants further studies. There are also studies of the effect of melatonin on cerebral arteries. Régrigny et al. (1999) found that melatonin can induce the increase of cerebral arteriolar tone via stimulating MT1 and/or MT2 receptors followed by blockade of calcium-activated large conductance potassium channels in rats, they also reported that melatonin decreased the lower limit of cerebral blood flow autoregulation, which may potential reduce the risk of hypoperfusion-induced cerebral ischemia. Later, Lapi et al. (2011) studied the rat pial microvascular responses induced by melatonin during brain hypoperfusion and reperfusion injury. They found melatonin can regulate the pial arteriolar tone and then promote an efficient redistribution of microvascular blood flow via activating MT1 and MT2 receptors, they further reported that lower dosage of melatonin stimulate MT2 receptors, while higher dosage activated also MT1 receptors (Lapi et al., 2011). From the above two studies, we can speculate that melatonin may have neuroprotective effect via regulating myogenic response of cerebral arteries.

### Melatonin Improves Cerebral Autoregulation by Ameliorating Hypoxemia and Regulating Metabolism

To ameliorate hypoxemia, mitochondrial function is crucial. Melatonin can protect mitochondrial functioning through its anti-apoptosis, antioxidative, and combined anti-apoptosis and antioxidative effects. Lopez et al. (2009) have indicated that melatonin protects mitochondria from damage due to oxidative stress by reducing oxygen consumption, membrane potential, and superoxide anion production. Carretero’s study presents the same conclusions (Carretero et al., 2009). Yamamoto and Mohanan concluded that melatonin protects against attenuated brain mitochondrial DNA damage induced by hydroxyl radicals (Yamamoto and Mohanan, 2002). In addition, Xu et al. (2016) found that melatonin potentials protects against cadmium neurotoxicity by blocking calcium-dependent translocation of Drp1 to the mitochondria. Recently, a study by Sinha et al. (2018) further reported that melatonin can inhibit mitochondrial cell death pathways by upregulating the MT1 receptor in newborn hypoxic-ischemic brain injury mice models. Besides improving the mitochondrial function, melatonin reportedly can act on cerebral nitric oxide/nitric oxide synthase after hypobaric hypoxia injury, which balances the release of nitric oxide, reduces peroxynitrite formation, and protects against nitrosative/oxidative damage (Blanco et al., 2017).

Thus, the collective general findings were that melatonin protects against hypoxemia. Although it is unknown whether melatonin can improve cerebral autoregulation after SAH by ameliorating the reduced brain-tissue oxygen pressure and brain pH, there is a theoretical basis for this hypothesis (**Figure 1**). It is worth mentioning that a study conducted by Herrera et al. (2014) found that, in chronically hypoxic lambs, melatonin improved vascular responses to potassium, serotonin, and methacholine and enhanced the endothelial response via nitric oxide-independent mechanisms in isolated arteries. This study indirectly indicates the possible impact of melatonin on cerebral autoregulation (Herrera et al., 2014).

### Comparison of Melatonin With Other Medications in Improving Cerebral Autoregulation

Previous studies have reported that several medications may have the potential to improve cerebral autoregulation after SAH. Nitric oxide plays an important in regulating cerebrovascular tone by maintaining the dilation of the vasculature. After SAH, nitric oxide production and responses to endothelium-dependent vasodilators were impaired owing to injury to the cerebrovascular endothelium, resulting in vasoconstriction (Sobey and Faraci, 1998). Consequently, nitric oxide (or nitric oxide donors) was proposed as a possible medication to improve cerebral autoregulation after SAH (Guo et al., 2016). In contrast to melatonin, nitric oxide improves cerebral autoregulation by activating calcium-dependent potassium channels in vascular smooth muscle, thus maintaining stable vascular tone after SAH.

Vasoactive substances can act on vascular smooth muscle, leading to cerebral arterial vasoconstriction or vasodilation. Some vasoactive substances, such as norepinephrine, adrenomedullin, and indomethacin, may have protective functions in cerebral autoregulation (Armstead et al., 2010a, 2016; Chock et al., 2012). However, no such protective function has been reported for other vasoactive substances, such as sodium nitroprusside (Armstead et al., 2010b; Baerts et al., 2013). It is notable that, unlike that for melatonin, the dose of vasoactive substances should be carefully monitored, as the impact of these drugs on cerebral autoregulation may vary based on the dose.

Additionally, pravastatin was reported has the function to improve cerebral autoregulation after SAH by improving vascular endothelium-dependent relaxation in response to acetylcholine, increasing endothelial nitric oxide synthase activity, and enhancing the vascular protective effects of Olmesartan (Yamamoto et al., 2007). These mechanisms have similarities and dissimilarities to the mechanisms of the action of melatonin. Additionally, we attempted to identify more antioxidants that have been reported to have effects on cerebral autoregulation, but failed to find any further evidence.

## Conclusion

Melatonin potentially impacts the control mechanisms of cerebral autoregulation after SAH through antioxidation, protection of endothelial cell integrity, suppression of sympathetic nerve activity, increase in nitric oxide bioavailability, mediation of the myogenic response, and amelioration of hypoxemia. Furthermore, melatonin may have a comprehensive effect on cerebral autoregulation after SAH.

## Author Contributions

Z-NG, HJ, HS, and YZ wrote the manuscript. JL and HM prepared the figures. XS and YY reviewed and edited the manuscript.

## Conflict of Interest Statement

The authors declare that the research was conducted in the absence of any commercial or financial relationships that could be construed as a potential conflict of interest.
